# Reduction Processes in Thin-Film Vanadium Oxides for Application in Optoelectronic Devices

**DOI:** 10.3390/nano16090528

**Published:** 2026-04-27

**Authors:** Dmitriy P. Sudas, Vasily O. Yapaskurt, Valery A. Luzanov, Galina G. Yakushcheva, Kirill Kuznetsov, Petr I. Kuznetsov

**Affiliations:** 1 Kotel’nikov Institute of Radioengineering and Electronics, Russian Academy of Sciences, Fryazino Branch, Fryazino 141190, Russia; 2 Faculty of Geology, Moscow State University, Moscow 119991, Russia; 3 Department of Quantum Electronics, Faculty of Physics, Moscow State University, Moscow 119991, Russia

**Keywords:** lossy-mode resonance effect, metal-to-insulator transition, MOCVD method, annealing process in vanadium oxides, VO_2_, V_2_O_5_

## Abstract

This article describes a study on the synthesis and annealing processes of thin-film coatings of vanadium oxide on flat, parallel substrates made of quartz glass, sapphire, and silicon, as well as optical fibers using an organometallic precursor, triisopropoxy vanadium (V) oxide. For the first time, optical constants of nanomaterials were estimated in real time during synthesis and subsequent annealed using the lossy-mode resonance effect. The coatings produced in an inert atmosphere after deposition were amorphous, comprising a mixture of VO_2_, V_2_O_5_, V_6_O_13_, and V_3_O_5_. This method allowed for accurate determination of the threshold temperature for the transformation of oxide mixtures into a monocomponent phase. Optimal conditions for synthesis and annealing were determined for the production of vanadium dioxide (VO_2_) and pentoxide (V_2_O_5_). Morphological changes in coated surfaces were observed as a result of heat treatment. The composition and properties of these samples were studied using optical, terahertz and Raman spectroscopy, as well as temperature-dependent analysis of electrical resistance. The morphology of the coating surface was determined using a scanning electron microscope and an atomic force microscope. The reduction of VO_x_ to VO_2_ was studied in an atmosphere of hydrogen and argon during annealing after deposition, with its effectiveness being compared. It was shown for the first time that the reduction of higher vanadium oxides is due to the presence of elemental carbon in the volume of the material formed from a metalorganic precursor during growth of vanadium oxide. Coatings obtained by annealing in hydrogen had a smaller hysteresis loop width (~5 °C) during phase transition compared to coatings obtained by argon annealing (~9 °C). Both types of coatings demonstrated a 50–60% increase in transmission at 1 THz frequency and in the IR region, accompanied by a 10^3^–10^4^-fold change in electrical resistance.

## 1. Introduction

Phase transitions are accompanied by effects of abrupt changes in material properties under the influence of external applied energy. At the same time, transitions can be classified into two categories: structural transitions [1], which involve changes in the crystal lattice, and electronic transitions [2,3], where only electronic properties change. Materials that combine both types of transitions are called Mott insulators [2,3,4]. Vanadium oxides have correlation properties of vanadium and are Mott insulators [4,5,6,7,8,9,10,11]. Depending on the ratio of oxygen and vanadium in the oxide, the increase in electrical resistance and the energy required for the transition to occur will differ significantly [12,13]. The temperature range of the transition occurrence ranges from cryogenic (−133 °C for V_2_O_3_) [14] to elevated (250 °C for V_2_O_5_) [15]. Since the structural transition is hysteresis, the shape and width of the loop depend on the morphology of the surface of the material and the crystallinity [16,17]. In general, the larger the individual crystallite, the narrower the loop width [16,18,19,20]. Vanadium dioxide with a transition near room temperature (~70 °C for VO_2_) has several polymorphic forms [21]. Among them, only VO_2_ (M) and VO_2_ (R) undergo a complete reversible metal-to-insulator transition (MIT). VO_2_ is considered the most promising for practical applications [22,23,24,25,26,27,28,29]. For any form and phase of vanadium dioxide with a structural transition, the characteristic critical temperature is in the range of 65–70 °C [24,25,26,27,29,30], while the width of the temperature hysteresis loop depends on the magnitude of the electrical resistance surge. When electrical resistance drops by 9000–30,000 times, the width is at least 5 °C [27], even at high temperatures, in order to obtain the material. It should be noted that the lowest values of separation between cooling and heating curves occur when resistance changes by 250 to 300 times [28]. Many applications are caused by changes in the optical constants of vanadium oxides during the transition, accompanied by a jump in the reflection. At the same time, the values of transmission variation vary significantly depending on the technology used to obtain the material and the precursor from which vanadium oxide was produced. The characteristic values for the infrared range of the spectrum are between 20% and 60% [24,31], while for the THz range, they are between 60% and 90% [16,28]. However, maximum values are achieved at high temperatures (over 700°C) after growth annealing. If we look at the measurement of the refractive index for vanadium dioxide, its characteristic value decreases by 0.2–0.3 after phase adjustment [28,30].

Depending on the method and conditions for the production of vanadium oxides, the resulting material will have different physical properties and MIT characteristics. Three main directions should be distinguished from the variety of methods for obtaining vanadium oxides [28,31,32,33]. The sol-gel method is relatively easy to implement, as it does not require high temperatures and vacuum, unlike magnetron sputtering. This makes it possible to obtain complex structures such as nanotubes and metastable phases of vanadium dioxide with outstanding MIT parameters [32]. In this case, the synthesis process often includes many intermediate stages and post-growth processing. Vapor chemical deposition makes it possible to obtain vanadium oxide coatings with varying crystallinity and surface morphology [20]. Moreover, the ability to transform material by post-deposition processes within a single technological synthesis cycle allows for the fine-tuning of the composition of resulting structures and their final properties. The main sources of vanadium in this method are reported in [34,35,36,37]. Metalorganic precursors are convenient sources of vanadium, as they have a high reactivity and a low decomposition temperature, making it possible to obtain coatings of high uniformity, even at atmospheric pressure. However, thermal decomposition processes and heterogeneous surface reactions lead to the formation of multiphase compounds with stoichiometries that differ from those of the deposited material.

In situ methods for controlling the composition of synthesized nanofilms using optical fibers are used in the creation of Q-switches for fiber lasers and in the production of sensors. The technique is based on the evaluation of the effect of deposited coatings and their optical characteristics on the transmission spectrum of geometrically modified silica fibers [38,39]. If the deposited material is optically transparent, with a real part of its complex refractive index greater than that of silica glass [39] and an imaginary part not zero, a lossy-mode resonance (LMR) will form in the transmission spectrum. The properties of this resonance under constant external conditions are dependent only on the coating thickness and its optical constants. Fiber refractometers are typically created using this principle [39,40]. However, each individual vanadium oxide has its own combination of refractive index and extinction coefficient in the transparency zone of silica glass. Therefore, studying the resonance shape directly during synthesis makes it possible to accurately assess both the composition and the threshold conditions for the transformation of the material during post-growth processing.

This paper describes an improved technique for evaluating annealing processes in the deposition of thin-film vanadium oxide using LMR in an optical fiber. The transformation processes of the composition of thin-film coatings of vanadium oxides during annealing are studied in detail. This paper is devoted to demonstrating a technique for rapid assessment of processes occurring in complex-composition coatings directly during synthesis and annealing. It also focuses on the effect of precursor on the final properties of coatings. Section 3.1 describes the principles of resonance formation, as well as its main characteristics and properties. A method for studying the annealing of vanadium oxides in a hydrogen atmosphere is shown in real time. Section 3.2 demonstrates the results of the synthesis of thin films obtained using metalorganic chemical vapor deposition (MOCVD) on sapphire and silica substrates, as well as their subsequent annealing in oxidizing and reducing environments. Section 3.3 shows the results of annealing processes in hydrogen and argon for deposited layers, as well as measurements of optical spectra for optical fibers and plates. The differences in the reduction mechanisms of vanadium oxides during annealing in reducing and inert atmospheres are shown. Section 3.4 is devoted to spectral evaluation of the grown coatings in visible and infrared regions, with the obtained samples examined using terahertz spectroscopy at a frequency of 1 THz.

## 2. Materials and Methods

**Figure 1:**
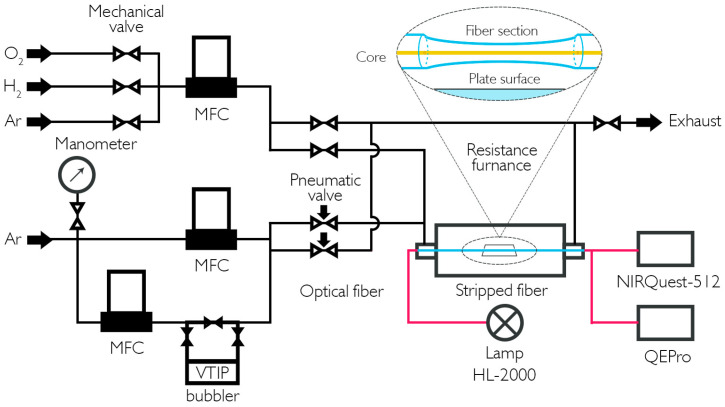
A diagram of a vanadium oxide coating setup with optical path transmission spectrum control is shown. The insert shows an enlarged image of a thinned segment of optical fiber.

The schematic of the MOCVD deposition system with in situ transmission control is shown in Figure 1.

The technique of forming thin-film coatings consists in the use of triisopropoxy vanadium oxide (VTIP) as a source of vanadium. It is placed in a stainless-steel bubbler and delivered to the reaction zone by argon. An additional Ar supply line controls the position of the deposition area inside the quartz reactor. During the process, the mass flow controllers at 50 cm/s set the linear velocity of the vapor–gas mixture. The bubbler containing the vanadium precursor is thermostated at 40 °C, while all gas lines and their mixing units are heated above 50 °C to prevent condensation. Deposition is performed in the temperature range of 180–300 °C using an annular resistance furnace to heat the tubular quartz reactor. Gas lines and pairs of pneumatic valves are used to compensate for pressure surges before each deposition or annealing experiment. Before each experiment, the tightness of the system is checked using a built-in pressure gauge. After the reagent flow rate was established, the flow was transferred to the reactor line, and an equivalent compensating gas was supplied to the discharge line. Some of the coating samples were additionally annealed in an atmosphere of argon, H_2_, or O_2_ to change the ratio of oxygen to vanadium in the coatings. The preformed films were heated to 255–465 °C and kept in a gas stream. Deposition and annealing were performed at atmospheric pressure (~1.5 atm). The flow rate of carrier gas through the evaporator was 550 cm^3^/min, and during annealing, gas flow was 300 cm^3^/min for both argon and hydrogen. After the growth stage, the reactor was filled with the necessary gas through a separate line and heated according to the program at various rates, ranging from 5 °C/min to 20 °C/min. After annealing, the reactor was cooled to room temperature in an inert gas atmosphere.

A chemically thinned fiber was placed in the reactor, together with plane-parallel sapphire or silica substrates. For this purpose, the optical fiber was stripped of its protective polymer coating over a 2 mm long section and treated with acetone to remove any residual impurities. A standard multimode optical fiber with a core diameter of 62.5 micrometers was then chemically etched, which almost completely removed the reflective coating. The etching process was carried out using a aqueous polishing solution of NH4F. Upon reaching the required thickness of 66 microns, the process was stopped, and the fiber segment was washed repeatedly with distilled water. The surface quality and geometry of the thin fiber segment were assessed and controlled using a optical microscope (NU-2 from Carl Zeiss AG, Oberkochen, Germany). The average rate of thinning using this etching method was 0.5 microns per minute.

The in situ transmission control measurement circuit used a halogen lamp (HL-2000 from OceanOptics Inc., Rochester, NY, USA) as the radiation source, and spectrometers (NIR Quest-512 and QEPro from OceanOptics Inc., Rochester, NY, USA) with an operating range of 400–1700 nm and a resolution of 0.1 nm served as receivers. The coating deposition rate was calculated based on its thickness, which was determined by scratch testing using an atomic force microscope AFM (NTEGRA Prima from NT-MDT LLC, Zelenograd, Russia). Due to their high degree of amorphism, it was not possible to evaluate the obtained coatings using X-ray diffraction XRD parameters (DRON -7 from NPP "Burevestnik", St. Petersburg, Russia). The main method for analyzing the composition of these coatings was the study of Raman scattering spectra obtained using an Raman spectrometer (Enspectr R532 from Spektr-M LLC, Moscow, Russia) λ = 532 nm, power = 30 mW, and spot size = 1 µm. Photographs of the surfaces were taken using a scanning electron microscope (JSM-6480 LV from JEOL Ltd., Tokyo, Japan). The transmission spectra of quartz and sapphire plates were obtained using a spectrometric complex (Specord UV-VIS from Carl Zeiss AG, Oberkochen, Germany).

Spot silver contacts at a distance of 1 mm from each other were applied to the surface of most coating samples, including those on optical fibers. The electrical resistance was measured by changing the ambient temperature from room temperature to 150 °C. The measurements were carried out in an automated chamber configured so that, at each set temperature, the deviation of the readings did not exceed 1%.

**Figure 2:**
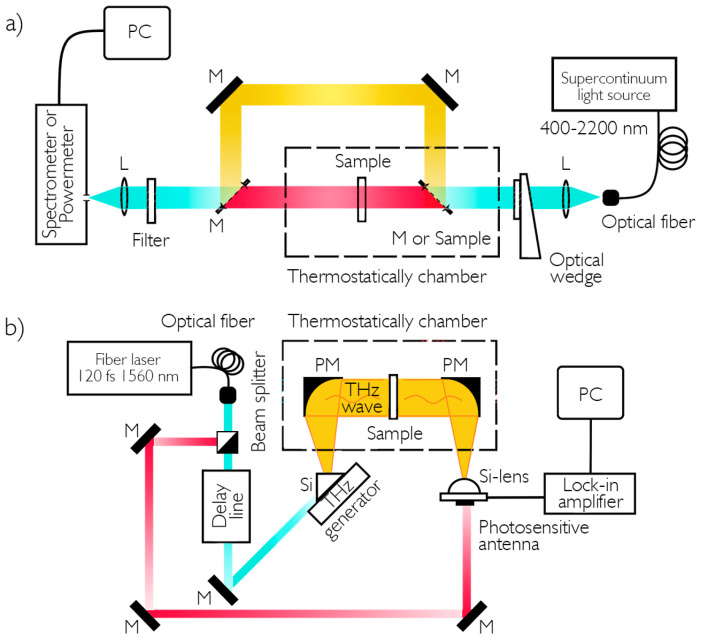
Schemes of spectroscopic studies of vanadium oxide coatings: (**a**) in the visible and near-infrared wavelength regions; (**b**) in the submillimeter wavelength region.

The spectrometric schemes for measuring the parameters of coatings are shown in Figure 2.

The measurement scheme in the visible and infrared spectral regions (Figure 2a) is a standard one for determining the transmission and reflection of samples. A fiber pigtail supercontinuum (Avesta-Project Ltd., Moscow, Russia) was used as a source of probing radiation. The spectrum width ranged from 400 to 2200 nanometers, with an average power of 30 mW. A set of lenses (L) with an anti-glare coating was necessary for uniform illumination of the test sample. A wedge was used to change the power of supplied radiation. In reflection studies, the sample was placed at an angle relative to the radiation and using several dielectric mirrors (M), and the reflected beam was directed to the entrance slit of an ASP-IR-2.6 spectrometer (Avesta-Project Ltd.). The scanning range of this spectrometer is 900–2600 nanometers, with a resolution of 0.1 nm. An power meter (PD300R-IR from Ophir Optronics Solutions Ltd., Jerusalem, Israel) was used in this scheme to study changes in absolute values of reflection and transmission at specific wavelengths. The temperature field was modulated in the chamber where the coating samples were placed.

Figure 2b shows the first experimental configuration for terahertz spectroscopy in the 0.1–1 THz range. A Mg:LiNbO_3_ crystal (Mg-8 mol.%) was used as a terahertz generator. The pump radiation was divided into two unequal parts in a ratio of 1:10 using a beam separator. Low-power radiation was directed to a photoconductive detector antenna based on a multilayer LT-InGaAs/InAlAs heterostructure formed on an InP substrate. Collimation of THz beam was carried out using two parabolic mirrors (PMs), between which a measurement unit was placed. After passing through the prism, the radiation was focused on a hemispherical lens of a photoconductive antenna, and the electrical signal from the antenna was amplified and recorded using an integrated amplifier.

## 3. Results

### 3.1. Using Lossy-Mode Resonance to Study the Annealing Processes of Thin-Film Coatings

For a preliminary assessment of steady-state growth processes, vanadium oxide coatings were grown on sapphire plates in an argon atmosphere at 180 °C. For example, the deposition rate at 180 °C was 0.42 nm/s, and when the temperature increased to 200 °C, it increased to 0.45 nm/s. The roughness of the samples obtained on an area of 1 μm^2^ was in the range of 10–12 nm. Ellipsometric measurements allowed us to obtain data on the dispersion of the complex refractive index of coatings obtained in the range of 400 to 1600 nm. VO_x_ films had n~2.2 in the region of 1300–1500 nm. Direct determination of stoichiometric composition using energy-dispersive X-ray diffraction proved impossible due to the overlap of oxygen and vanadium peaks. Since the obtained coatings showed no signs of a phase transition at a temperature of about 70 °C, it was necessary to determine the annealing conditions for converting them into a material with a VO_2_ phase content that demonstrates such a rapid transition. A full description of the composition and transformations occurring in the coatings during annealing will be given in Section 3.2.

Upon deposition on a thin fiber, when a certain VO_x_ layer thickness was reached, an LMR formed in the transmission spectrum. As it continued to grow, it moved to a longer wavelength region of the transmission spectrum, then disappeared from view. Subsequently, as the coating thickness increased, another LMR was formed in the spectrum. When the maximum LMR reached the range of 1300–1400 nm, growth stopped. The material deposited on the fiber had a thickness of ~170 nm, which coincided with the thickness of the coating on the sapphire substrate. Tests of the LMR-based technique were performed at thicknesses of no more than 170 nm. This thickness was chosen to ensure sufficient continuity of the coating where the first resonance order forms. In such conditions, the resonance depth is maximized and is easy to analyze. At the same time, with this thickness, a plateau of the maximum amplitude of electrical resistance surge is reached, and several microns are retained. According to our preliminary experiments and the literature, there is evidence that phase-transition parameters in vanadium oxide coatings significantly change at thicknesses of less than 100 nm [35]. To form a resonance, the real part of the refractive index of the surrounding medium must be less than that of silica glass and the vanadium oxide coating, and the imaginary part must be greater than that of adjacent materials. The extinction coefficient of the coating must have a low value but not be zero [39]. This criterion suggests that the deposited material does not contain a predominant amount of vanadium dioxide. Since literature data [41] states that the extinction coefficient is greater than the refractive index in the IR region for VO_2_, LMR is not expected to appear. It should also be noted that dioxide is in a metallic state due to the synthesis temperature. Since composition is not precisely defined at this stage, the coating is called VO_x_.

To evaluate the transformations occurring in coatings, we use a theoretical model to describe the lossy-mode resonance in a segment of thin optical fiber. The cross-section of the etched section of an uncoated fiber optic cable is represented by a Fabry–Perot interferometer. An isotropic coating with a complex refractive index of n_2_ is located between two semi-infinite layers with lower refractive indices of n_1_ and n_3_ (n_2_ > n_1_ ≥ n_3_). n_1_ and n_3_ are assumed to be the refractive indexes of silica glass and the environment. Diffuse reflection can be ignored because the amount of roughness on the silica surface (SiO_2_–VO_x_ interface) is negligible over the wavelength range under consideration. The basic equation takes the following form [42]:

(1) $$T={\left[\frac{1}{2}{\left|\frac{r_{s1}+r_{s2}exp(iϭ)}{1+r_{s1}r_{s2}exp(iϭ)}\right|}^{2}+\frac{1}{2}{\left|\frac{r_{p1}+r_{p2}exp(iϭ)}{1+r_{p1}r_{p2}exp(iϭ)}\right|}^{2}\right]}^{N},$$

where r_s1_ and r_p1_ are the Fresnel coefficients for the SiO_2_–VO_x_ boundary and r_s2_ and r_p2_ are those for the VO_x_–air boundary. The first part of the sum contains the contribution of perpendicular polarization, and the second part contains the contribution from parallel polarization. N is the number of reflections at the SiO_2_–VO_x_ interface, depending on the length of the thin section, its diameter, and the angle of incidence at the interface. ϭ is the phase delay caused by the difference in the optical path of rays passing through the coating to the environment and depends on its thickness (d).

A minimum of optical transmission (resonance) will be observed propagating through a dielectric waveguide when the cut-off conditions for a mode supported by a thin layer of VO_x_ coating are met [43]. Then, the criteria for clipping the coating thickness at a certain wavelength for any order of TM mode in such a structure are

(2) $$d_{m_{LMR}TM}=\frac{\lambda }{2\pi {\left(n_{1}^{2}-n_{2}^{2}\right)}^{\frac{1}{2}}}\left[arctan\left[{{\left(\frac{n_{1}}{n_{3}}\right)}^{2}\left(\frac{n_{2}^{2}-n_{3}^{2}}{n_{1}^{2}-n_{2}^{2}}\right)}^{\frac{1}{2}}\right]+m\pi \right].$$

**Figure 3:**
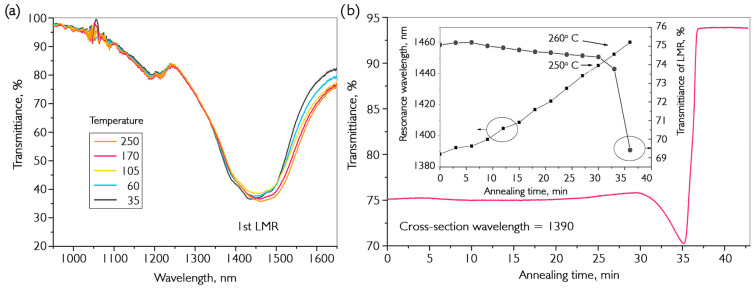
Results of the synthesis of VO_x_ coating on the surface of an optical fiber. (**a**) A typical transmission spectrum of a fiber path with deposited film at different argon temperatures inside the reactor. (**b**) A cross-section of the transmission spectrum at a wavelength of 1390 nm for a sample coated with 170 nm thick film during annealing in hydrogen; the insert shows dependences of resonance position and depth on annealing time.

Unlike metals, resonances in semiconductor coatings are formed when the fundamental mode of the optical fiber interacts with the transverse electric (TE) and transverse magnetic (TM) components of the coating. For each order of LMR, there is a pair of resonances named after the TE and TM components. The first of the obtained pairs of dips is the resonance caused by the interaction of the fundamental mode with the TE component of the coating, since the required cut-off conditions for this component are met at a shorter wavelength than those for the TM component. The focus is on describing the first TM LMR, as most studies have been conducted using this resonance. As previously mentioned, the first TE LMR appears at a thickness where irregularities in the continuity of the coating cause a distortion in the shape of the dip. It is possible to calculate the required film thickness for observing resonance in a selected area using information about the type and order of resonance, as well as the refractive indices of the coating, fiber, and environment. In addition, changes in the shape and position of LMR in the transmission spectrum of fiber optics can be traced back to changes in the coating during annealing. A more detailed theoretical description of resonance is given in references [38,42,43]. Figure 3 shows data on the transmission of one series of VO_x_ coatings deposited on an optical fiber at 200 °C, followed by annealing in a hydrogen atmosphere. The temperature conditions cannot be determined from the reference data because the material is a complex mixture of vanadium oxides. Therefore, a series of preliminary experiments was conducted to determine the precise temperature conditions.

Preliminary tests showed that, in the temperature range up to 250 °C, there is a reversible shift of the resonance to a longer wavelength region (Figure 3a) caused by thermal expansion of the coating. The magnitude of the LMR displacement in the transmission spectrum with a temperature change is 20 nm/100 °C. For temperature testing during annealing, a series of samples was specially created with maximum resonance at 1350 nm. A critical point was observed in the reduction medium (inset in Figure 3b) above which the resonance began to rapidly transform with linear heating from room temperature. At different heating rates of 5–20 °C/min at 250 °C, a slight increase in the depth of resonance was observed, accompanied by its shift to shorter wavelengths. At the same time, there was a slight delay between an increase in resonance depth and the beginning of displacement. Presumably, this was caused by a lag in the transformation of the surface layers of the deposited coating and its volume due to the heterogeneity of the material. It is worth noting that long-term annealing (several hours) at temperatures below the critical temperature does not lead to a noticeable change in the shape of resonance.

**Figure 4:**
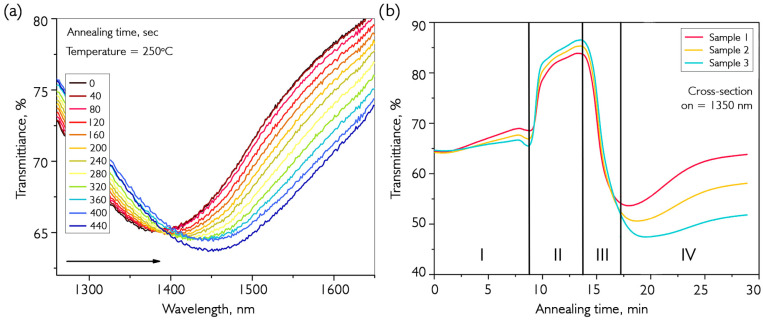
Spectral characteristics of the fiber path during annealing of the VO_x_ coating. (**a**) The transmission spectrum of an optical fiber with a deposited film during annealing in hydrogen. (**b**) The cross-section of the transmission spectra at a wavelength of 1350 nm for several samples during their annealing in H_2_.

The next stage of the study involved annealing at a fixed temperature. The sample with the deposited coating was preheated to 250 °C; then, hydrogen was supplied to the chamber and maintained for an hour. The results of the annealing transmission measurement are shown in Figure 4.

**Figure 5:**
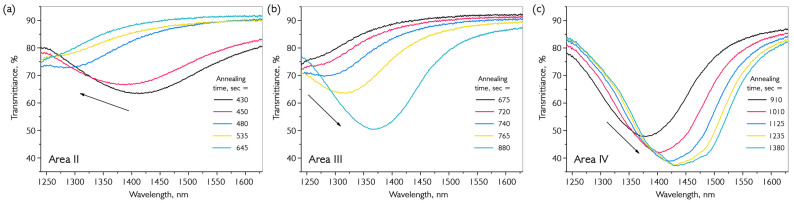
Change in the transmission spectrum of a VO_x_-coated fiber path during annealing at 250 °C in a hydrogen atmosphere in the time following zones: (**a**) II; (**b**) III; (**c**) IV.

Figure 4a shows the first stage of heating until the critical temperature is reached and hydrogen is introduced into the chamber (at 400 s). After the argon atmosphere has been replaced by hydrogen, there is an increase in resonance depth and a further active shift towards the long-wavelength region, as shown in Figure 3. This annealing process is accompanied by several distinct resonance transformations that are clearly visible in cross-sectional transmission spectra (λ = 1350 nm), as shown in Figure 4b. There are four distinct periods with different transmission patterns during hydrogen annealing: I—a slight change in transmission during heating to the annealing temperature and hydrogen injection; II—a sharp increase in transmission due to a shift of the resonance wavelength to a shorter region with a reduction in its depth; III—repeated movement of the resonance into the long-wavelength region with a sharp increase in depth and its further transition beyond 1350 nm; and IV—continuation of the movement of LMR and stabilization. The complete change in transmission spectrum in time sections II, III, and IV at an annealing temperature of 250 °C is shown in Figure 5.

**Figure 6:**
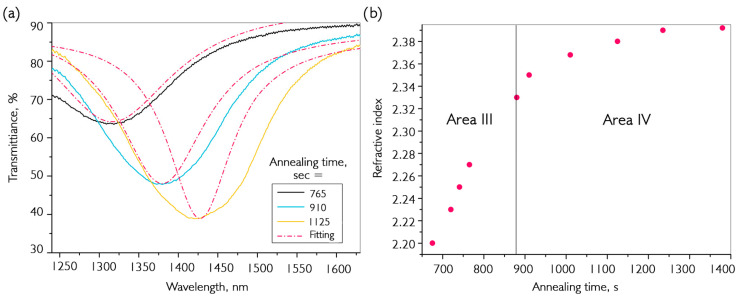
Determination of the refractive index of thin-film vanadium oxide during annealing in hydrogen: (**a**) comparison of the shape of the transmission spectra of the fiber path obtained using a theoretical model and an experimental data; (**b**) dependence of the refractive index of the coating on the annealing time in hydrogen.

It is possible to interpret the processes occurring in annealed coatings by having a theoretical model of LMR and by knowing what factors affect its behavior in the transmission spectrum. In zone II, there is a phase restructuring of the material with an increase in its crystallinity degree. As a result, the material becomes more compact and occupies less volume, and its thickness decreases. This effect has also been described during the annealing of vanadium oxides in a reducing atmosphere [44]. Using ellipsometric measurements to determine the refractive index of grown material, we calculated that such displacement leads to a reduction in coating thickness of approximately 8–10%. By stopping the annealing process at this stage and using AFM, we determined that the coating had, indeed, decreased in thickness to the calculated value. According to literature data, the effect of thermal decomposition is not observed at such low temperatures. Regions III and IV combine to form a similar resonance behavior, with motion in the long-wavelength part of the spectrum. It is important to note two factors: the first is that the thickness of the material does not increase and even continues to decrease slightly; the second is that the depth of the maximum LMR at 1400 nm in zone III is significantly greater than in zone II. This is caused by an increase in the real part of the refractive index of the coating (Equation (1)). It is possible to calculate the magnitude of this change using a theoretical model. An estimate of the refractive index during annealing of a thin-film coating is shown in Figure 6.

The experimental resonances shown in Figure 6a have a greater width than the theoretical ones. This is easily explained by the fact that LMR models are formed only when interacting with the fundamental mode of fiber, and experimental data were obtained using multimode fibers. According to the performed calculations, during annealing, the value of the refractive index abruptly increases from n ~2.2 to 2.35, then slowly reaches saturation and stops near 2.39. Further annealing at this temperature does not significantly change resonance parameters. An increase in the refractive index occurs due to conversion of VO_x_ into VO_2_ material, whose refractive index in the λ = 1300–1500 nm range is greater than 3. In this case, reduction occurred partially, which suggests that annealing should be performed at higher temperatures. Additionally, it is worth noting that when n stabilizes, the resonance maxima begin to blur. This indicates the appearance of an additional phase with a different composition in the material. Summarizing the obtained results, we can say that the deposited material is a mixture of several phases of vanadium oxide, and its reduction by hydrogen to VO_2_ depends primarily on the temperature. After conducting similar experiments at higher temperatures, we determined that above 350 °C, the resonance disappears due to the emergence of a material with a high extinction coefficient, which may be a marker for the predominance of vanadium dioxide in the composition. In the near-infrared spectral region, this material has an imaginary part of the complex refractive index greater than the real part.

### 3.2. Production of Pure Phases of Vanadium Dioxide and Pentoxide

**Figure 7:**
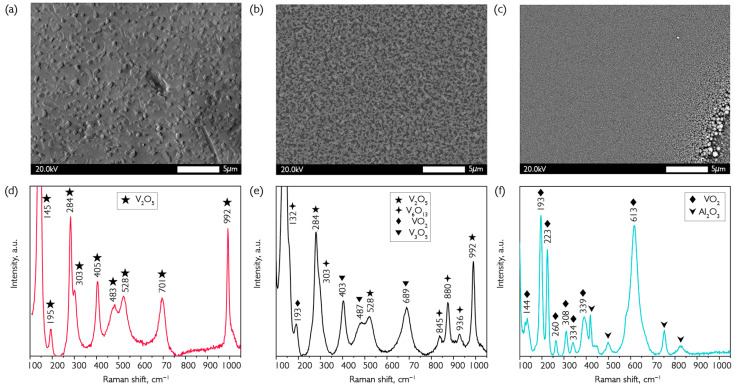
Determination of the composition of VO_x_ coatings obtained after deposition (**b**,**e**) and annealing in oxygen (**a**,**d**) and hydrogen (**c**,**f**). (**a**–**c**) SEM images of the obtained coatings; (**d**–**f**) Raman spectra. All the samples were obtained on sapphire substrates. The thickness of the deposited sample was 1750 nm.

As mentioned earlier, standard methods for determining the composition of vanadium oxide coatings are not applicable, so there are only three ways left: the direct method—by studying Raman spectra—and two indirect methods—by studying transmission spectra and the position of the temperature point of the phase transition. The latter method requires the application of contacts to the surface of the material, so further heat treatment of such samples may lead to unreliable results. The LMR method was used to preliminarily determine deposition and annealing conditions. The results of the characterization of the obtained coatings, including those subjected to subsequent annealing in oxygen and hydrogen, are shown in Figure 7. All coatings were obtained at a temperature of 200 °C. Different substrates were used for a more comprehensive analysis of the synthesized materials. Depending on the composition and orientation of the substrate, the crystallinity of the grown material can vary significantly. The LMR method described in this paper requires silica fiber as a substrate. SiO_2_ plates were also used to estimate optical constants and transmission variations. Raman spectra have more pronounced peaks on sapphire substrates, while on quartz plates, the location and relative intensity of peaks coincide with those obtained on Al_2_O_3_. For EDX analysis, silicon plates are required. Since the coatings obtained using the MOCVD method at relatively low temperatures (less than 500 °C) are amorphous, X-ray diffraction analysis does not allow reliable data to be obtained. With these substrates, oxygen present from the plate does not interfere with the assessment of the amounts of vanadium and oxygen in the deposited material, so various plates are used to demonstrate analysis data of grown coatings more clearly. At the same time, multiple experiments have confirmed the repeatability of vanadium oxide compositions on different substrates under the same synthesis conditions.

Considering the coatings obtained immediately after synthesis, several phases with characteristic peaks in the Raman spectrum can be seen at once: V_2_O_5_ (284, 528, and 992 cm^−1^), V_6_O_13_ (132, 303, 845, 880, and 936 cm^−1^), VO_2_ (193 cm^−1^), V_3_O_5_ (403, 487, and 689 cm^−1^) [45]. Moreover, V_2_O_5_ and V_6_O_13_ are dominant in quantitative terms. All three coatings were obtained at a synthesis temperature of 200 °C, with a resulting thickness of 1750 nm. The thickness was determined using a scratch on an atomic force microscope. The photo shows that the coating has a granular structure with differing crystallinity. After annealing in oxygen for 15 min at 300 °C, large crystallites combine into larger coatings that push onto the surface. This is due to oxidation of the surface layers and the locking of oxides with lower vanadium oxidation degrees. Similarly, only lines characteristic of V_2_O_5_ are visible in the Raman spectrum after annealing at 350 °C for 15 min. The coating becomes more uniform after annealing in hydrogen at this temperature because of lower porosity due to depletion of oxygen. The substrate line in the Raman spectra also indicates higher uniformity. Annealing was performed in oxygen at a temperature of 300 °C to convert the material into higher vanadium oxide (V). The technique was the same for hydrogen annealing and oxygen annealing. When the set temperature was reached, the reactor was filled with oxygen and maintained for 30 min, then cooled. Presumably, transformations occurred according to the following equation:

(3) $$2V_{6}O_{13}+2VO_{2}+2V_{3}O_{5}+5O_{2}=10V_{2}O_{5},$$

After the oxidation reaction, only vanadium pentoxide lines were visible in the Raman spectrum of the coating at 145, 195, 284, 303, 405, 483, 528, 701 and 992 cm^−1^. The morphology of the oxidized surface underwent a significant change, and individual large crystallites with an average size of about 500 nanometers began to form (Figure 7a).

To convert a multiphase coating of oxides with a high percentage of vanadium in the oxidation state (+5) to vanadium dioxide in the metal oxidation state (+4), we used hydrogen at a temperature of 350 °C for 15 min of annealing. The overall reduction reaction of the oxides detected by Raman spectroscopy in the grown coatings to VO_2_ can be expressed as follows:

(4) $$V_{6}O_{13}+V_{2}O_{5}++V_{3}O_{5}+H_{2}=11VO_{2}+H_{2}O,$$

**Figure 8:**
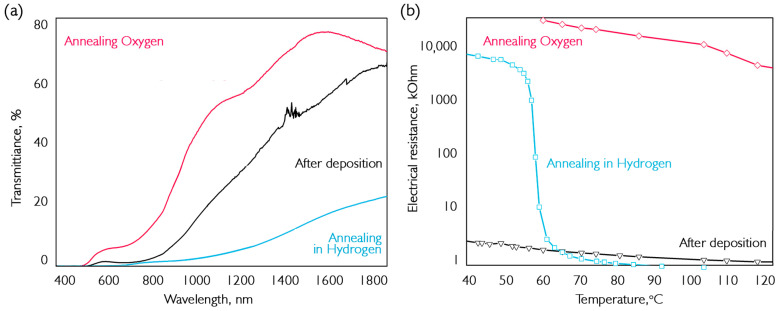
Determination of the composition of VO_x_ coatings: (**a**) optical transmission; (**b**) dependence of electrical resistance on ambient temperature. The thickness of the deposited sample is 250 nm.

Incomplete removal of the resulting water from the coating leads to a noticeable content of OH groups, which is a negative factor in optics. The fact that recovery takes place completely within 15 min was repeatedly confirmed by Raman spectra, in which only VO_2_ lines are present: 144, 193, 223, 260, 308, 334, 339 and 613 cm^−1^. Due to the limited thickness of the recovered coating, probing radiation reaches the substrate, and sapphire lines are visible in Figure 7f. After reduction, crystallites decrease in size, and the coating becomes more uniform (Figure 7c), as mentioned previously. Additionally, coatings were evaluated based on the position of the phase transition and absorption edge in transmission spectra. The results of these measurements are presented in Figure 8.

According to literature data, the absorption-edge wavelength of vanadium dioxide is approximately 1500 nm, while that of pentoxide is almost in the visible region [46]. The change in absorption edge is primarily due to the appearance of predominant VO_2_ or V_2_O_5_ phases. The initial thickness of the deposited layer is about 250 nm and varies by no more than 10% due to annealing. The dependences on the nature of electrical conductivity (Figure 8b) show that the annealed sample has a gigantic electrical resistance. Because the transition of this oxide occurs above 250 °C, the material is in an insulating phase in this region, which explains its resistance. The annealed material in hydrogen is vanadium oxide with a phase transition at 68–69 °C and an electrical resistance increase of 9700 times.

The technique of annealing films in hydrogen to reduce VO_2_ is understandable, but as a result, coatings contain a contaminating component in the form of water.

### 3.3. The Participation of Carbon in the Reduction of Vanadium Oxides 

**Figure 9:**
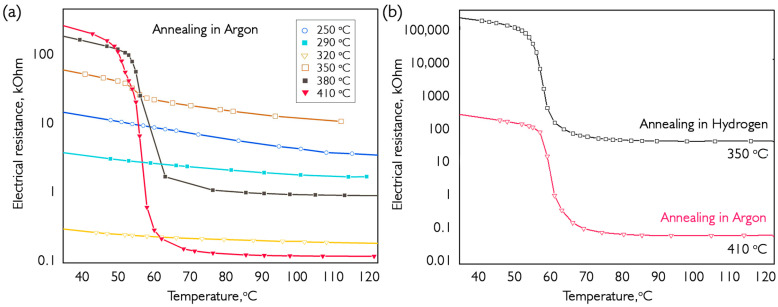
Comparison of the temperature dependences of the electrical resistance of VO_x_ samples: (**a**) annealed in argon at various temperatures; (**b**) annealed in a mixture of hydrogen and argon. The graphs show data for samples cooled from 120 °C. The thickness of the deposited sample is 250 nm.

To determine the processes occurring during annealing in an inert atmosphere of VO_x_ composite coatings, a series of tests was conducted in argon at temperatures ranging from 250 to 410 °C. The annealing time for all processes was 15 min. The results are shown in Figure 9.

The temperature dependence of electrical resistance in Figure 9 shows that as the annealing temperature increases, a jump occurs on the curves associated with the phase transition in vanadium dioxide. The data on the amplitude of the electrical resistance surge in samples obtained by annealing in argon and hydrogen are shown in Table 1. R_(40)_/R_(90)_ is the ratio of electrical resistance at temperatures of 40 and 90 °C.

Raman studies have shown that annealing between 250 and 320 °C leads to a decrease in the V_2_O_5_ phase and an increase in V_6_O_13_. Since the MIT occurs at cryogenic temperatures, the electrical resistance of the initial coating decreases by a factor of 20 after annealing at 320 °C. At 350 °C, it increased by two orders of magnitude, and a jump was noticed on the cooling curve at about 55 °C. This was due to the formation of a significant amount of VO_2_ in the coating. Increasing the annealing to 410 °C allowed us to achieve, according to Raman spectroscopy, an almost pure VO_2_ state. This resulted in a 3000× increase in electrical resistivity. For a direct comparison of the annealing results for hydrogen and argon, the temperature was increased until the amplitude of the electrical resistance jump became equal. Figure 9b shows a 3000× increase in electrical resistance for vanadium dioxide coating samples prepared using two different post-treatment methods. Two facts should be noted: During annealing in argon, a higher temperature is required, but the resulting dependence exceeds the total electrical conductivity of a similar sample prepared during annealing with hydrogen. The three-order-of-magnitude lower position of the whole temperature dependence during argon annealing indicates the significant presence of vanadium oxide in the composition of the coating located in the metallic phase at temperatures under consideration. Based on phase diagrams [16] and MIT parameters, V_6_O_13_ is the only compound that fits this criterion. Since another oxide, besides VO_2_, is still present in the coating volume but does not adversely affect the properties of the MIT, this impurity is present in a separate crystallite volume and does not interfere with structural changes during the transition.

**Figure 10:**
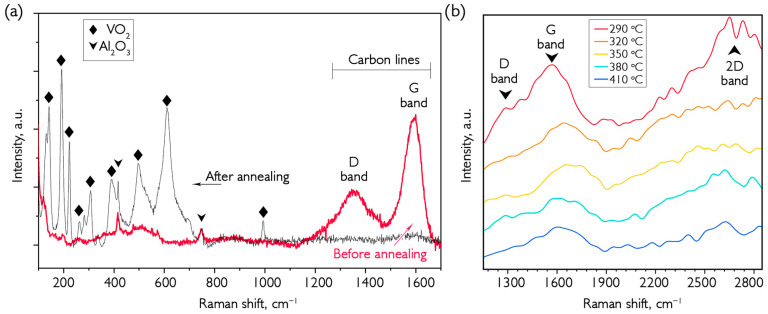
Raman spectra of VO_x_ coatings obtained at 300 °C and annealed in argon: (**a**) the spectrum before and after annealing in the range of 100 to 1700 cm^−1^; (**b**) the spectrum at various annealing temperatures in the range of 1200 to 2800 cm^−1^. The thickness of the deposited sample is 1670 nm.

To determine the mechanism of conversion of the VO_x_ coating to VO_2_ during annealing in an inert argon atmosphere, a sample was grown at a temperature of 300 °C. The results of the Raman study of the sample after deposition and annealing in an argon atmosphere at different temperatures are shown in Figure 10a,b.

It can be seen that the graphs show a wide band at about 2600 cm^−1^, which is usually labeled as the 2D band of graphite and is linked to graphene. The number of graphene layers is usually determined by intensity and shape [47]. At the same time, the line has a low intensity (compared to the line for pure graphene) due to the absence of a pronounced layered carbon structure in the material. A G-line can be seen at about 1580 cm^−1^, associated with the stretching of the graphite lattice, reflecting the degree of order in the structure. Moreover, this peak consists of two parts, fused with the D-line at 1350 cm^−1^, which appears due to defects and disorder in the carbon structure. As the annealing temperature increases, both these lines disappear, as well as the 2D line. This decrease in carbon in the surface layer is due to formation of carbon dioxide and the deposition of material in an argon atmosphere during high-temperature annealing. The higher the annealing temperature, the less carbon remains in the lattice, and the corresponding lines in the Raman spectrum become less intense. The initial structure of carbon in the lattice is similar to black carbon and has the highest degree of crystallinity. After annealing, only a line at about 1600 cm^−1^ remains, which arises from the C=C bond and indicates incomplete disappearance of carbon from the material.

After deposition, the bands associated with vanadium oxides are weakly pronounced in the Raman spectrum, and narrow peaks of low intensity on the sapphire substrate are visible. Blurred, weakly visible bands from vanadium oxide indicate a high degree of amorphous oxide coating. It should be emphasized that carbon lines of such high intensity are only observed in the Raman spectra of samples deposited at unusually high temperatures. The standard temperature range for VO_x_ deposition is 180–200 °C, which ensures the growth of higher-quality materials with well-defined vanadium oxide peaks, while carbon lines are weak. As the annealing temperature increases, carbon line intensity gradually decreases and, conversely, oxide phase peaks begin to resolve and become more intense. Figure 10a shows the spectrum in the frequency range of 150–1650 cm^−1^ after final annealing at 410 °C. The position of the oxide peaks correlates well with the VO_2_(M) phase, and only a faint trace of carbon lines remains at 1600 nm. We note three experimental facts: 1—for the first time, it was possible to detect the presence of carbon in deposited coatings of vanadium oxides; 2—carbon participates in reducing processes in coatings made from a mixture of V_2_O_5_ and other oxides with mixed +5 and +4 valences of vanadium; 3—the reduction process of such coatings can be stopped by the formation of an almost pure VO_2_(M) phase.

The carbon content in the deposited material clarifies the mechanism of reduction of higher vanadium oxides (V_2_O_5_ and V_6_O_13_) in the coating to form oxides with reduced vanadium valence—in particular, the VO_2_ phase. The equation according to which this reduction process is carried out is

(5) $${2V}_{6}O_{13}+{2V}_{2}O_{5}+2V_{3}O_{5}+C=22VO_{2}+{CO}_{2}$$

**Figure 11:**
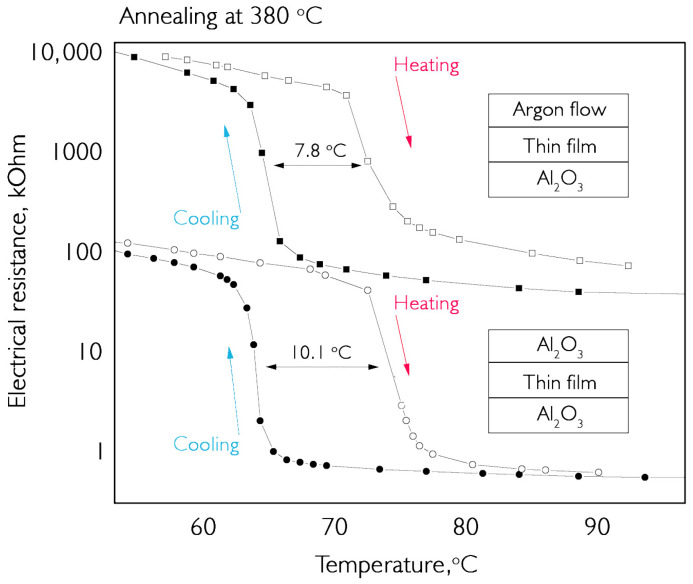
Comparison of electrical resistance hysteresis loops in two vanadium oxide coatings annealed in an argon atmosphere: (**top**) in contact with an ambient gas stream; (**bottom**) covered with a sapphire plate. The inserts show a schematic of the layers during annealing. The thickness of the deposited samples is ~200 nm.

To avoid contamination of the argon medium, an experiment was conducted to test the annealing of two identical coatings with and without a carrier gas stream. The first sample was annealed in argon at a temperature of 380 °C, and the second sample was sandwiched between two sapphire plates. The results of the comparative tests are shown in Figure 11.

The results show that, during the same annealing time, a sufficient amount of vanadium dioxide is formed in both samples to maintain the same transition amplitude. At the same time, the coating sample sandwiched between two sapphire surfaces has a similar resistance jump of ~200 times, while the overall resistance level is several orders of magnitude lower. This higher conductivity indicates the presence of a certain amount of phase in the composition that has a presence in the cryogenic region. Such an impurity at temperatures near room temperature arrives in the metallic phase and reduces the overall resistance of the sample. V_6_O_13_ and V_2_O_3_ have transitions in the range of 140–160 K but contain significantly different amounts of oxygen molecules. Based on studies of Raman spectra, it was determined that the composition lacks the characteristic peaks associated with V_6_O_13_. At the same time, the overall pattern of the spectra indicates a high degree of amorphousness in the obtained materials. No reliable data on V_2_O_3_ is available in the literature, but peak blurring due to scattering is observed in all samples. In addition, the hysteresis loop width is smaller for samples in contact with argon. This is attributed to greater uniformity and lower porosity of the coatings. All this suggests that carbon is present in the deposited material and that it is consumed during annealing, releasing carbon dioxide. During annealing of coated samples, sapphire interferes with the free release of reaction products, leading to the accumulation of gas molecules in the lattice and their stretching.

The reaction of the formation of vanadium oxide from vanadium precursor OV(OCH(CH_3_)_2_)_3_ is heterogeneous and occurs on the surface of the substrate. A frequently used method is the density functional theory (DFT), which is used in computational chemistry and does not fully describe the processes occurring with vanadium precursors at the stage of coating synthesis [34]. However, this method has some limitations, especially when working with vanadium precursors and vanadium oxides in general. In this paper, we have focused on a more applied description of the proceeding processes. In the ongoing rearrangement reaction, V_2_O_5_ and complex hydrocarbon molecules are formed, which are removed from the reaction zone by argon. In this case, there is no change in the valence of carbon and vanadium, but carbon-containing products and the precursor, itself, can be trapped in the pores of the coating. It is more energetically advantageous for one detached radical to lock onto an identical or similar one [34]. At the same time, only free carbon, which is not bound to other atoms, is visible in the Raman spectrum. During precipitation at higher synthesis temperatures, thermal decomposition of the precursor in the gas phase occurs partially. The hydrocarbon radicals formed in this case in an inert atmosphere are forced to enter into disproportionation reactions. These reactions eventually form complex hydrocarbon molecules with C=C double bonds and elemental carbon that settle on the surface of the grains in the growing film.

To confirm the participation of accumulated carbon in the coating in reduction processes, a series of sequential annealing was performed in argon and oxygen. The sample was annealed at 400 °C in argon, then oxidized in oxygen until only V_2_O_5_ lines were visible in the Raman spectrum. After that, the coating was annealed again in an inert atmosphere under the same conditions as the first annealing. A similar cycle was repeated five times. As a result, the amount of vanadium dioxide formed during annealing in argon consistently decreased. This indicates depletion of carbon reserves in the coating volume.

### 3.4. Characterization of Filters on Thin VO_2_ Films for IR Wavelength Range and Terahertz Frequency Radiation

**Figure 12:**
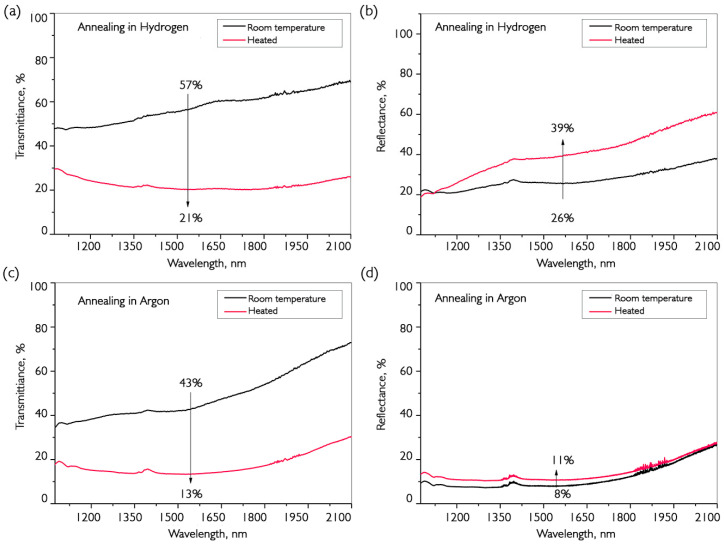
Optical transmission (**a**,**c**) and reflection (**b**,**d**) spectra before and after the phase transition in VO_2_ films on sapphire obtained by annealing in hydrogen (**a**,**b**) and argon (**c**,**d**). The thickness of the deposited samples is ~190 nm.

Optical filters are one of the main applications of vanadium oxide coatings. Additionally, recently, in the field of medical research on THz sources, it has been found that the modulation of the transmission of VO_2_ coatings allows them to be used for selective filtering of submillimeter waves. Two methods for producing VO_2_ films were demonstrated in previous sections and tested for their optical characteristics in the near-infrared region. Experimental results are shown in Figure 12.

The coating samples were synthesized on sapphire and annealed in such a way as to ensure the same amplitude of the electrical resistance jump at the VO_2_ phase transition. In addition to the spectrometer, an optical power meter and three laser radiation sources at wavelengths of 976, 1238 and 1548 nm were used to determine the absolute values of the transmission change.

The graphs in Figure 12 show the transmission and reflection spectra at room temperature and above the phase transition temperature (~90 °C). For comparison, Table 2 shows the characteristics of the transmission spike in samples at different annealing temperatures. The thickness of the deposited samples is ~190 nm. In all experiments, the annealing time at each temperature was 10 min.

It can be seen that maximum values of transmission change are not observed at the maximum amount of vanadium dioxide. This is presumably caused by the need for the presence of vanadium oxide crystallites in a larger matrix, like V_6_O_13_. Therefore, structural restructuring occurs over a larger volume of material.

**Figure 13:**
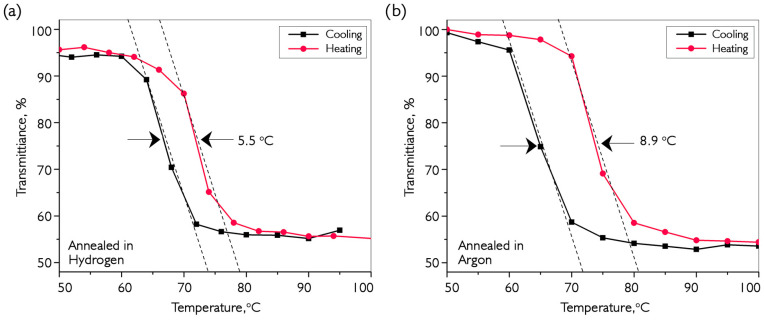
Investigation of transmission dependencies for VO_2_ coating samples annealed in hydrogen (**a**) and argon (**b**) at different ambient temperatures for waves with a frequency of 1 THz. The thickness of the deposited sample is 190 nm.

It can be seen that the transmission jump is approximately the same for both variants of annealed samples, while the increase in reflection for coatings annealed in hydrogen is significantly greater. However, the situation is different for the reflection spectra, where the increase in argon-treated coating reflection is negligible. Therefore, in this type of sample, the main growth occurs in the scattering region during the transition. Moreover, it is clear that after annealing in hydrogen, the film quality is better, as the transmission before the transition was 57%, compared to 43% using the alternative production method. Additionally, Figure 13 compares the hysteresis of the transmission jumps for submillimeter wavelengths.

The samples selected for the study after annealing in hydrogen and argon demonstrate the amplitude of the transmission change during the phase transition in VO_2_ at the level of results given in the literature [8,28]. However, it should be noted that we did not find published data with similar characteristics for coatings deposited by CVD in an inert atmosphere using the precursor we used. The coatings obtained as a result of the work demonstrate changes in transmission in the IR range at a level of 60%, which exactly coincides with the results of other authors [28,30,34]. At the same time, for the first time, coatings using VTIP demonstrate a 55% increase in transmission at a frequency of 1 THz. The current maximum value for this frequency using the sol-gel method and post-growth annealing is 90%. Generalization of data on the changes in transmission of coatings annealed in H_2_ and Ar during phase transitions to VO_2_ revealed that the main difference lies in porosity and crystallinity. Samples annealed in hydrogen are more homogeneous and transparent, while coatings annealed with Ar have higher porosity, allowing for modulation of scattering with the same amplitude variation in transmission at the MIT. The stored carbon from the organic molecule, upon exiting the volume of the material, leads to dislocations and lattice defects that cause different absorption behaviors before and after the phase transition. In addition, the required temperature for the formation of the required amount of vanadium dioxide at the grain boundary is significantly higher than during annealing in hydrogen, which, in turn, negatively affects the uniformity of crystallites and the width of the temperature hysteresis of the phase transition. In addition to having higher conductivity, such coatings are also more preferable as filters, since they will not produce additional back reflection in laser circuits. We obtained preliminary results from using vanadium oxide coatings as Q-factor modulators in fiber laser resonators [48]. At the same time, many experiments are still needed to compare the efficacy of these coatings as filters. It is worth noting that the absorption peak at a wavelength of about 1380 nm, associated with the content of OH groups, is 3.7 dB higher for the coating annealed in hydrogen than for the coating after annealing in argon

## 4. Conclusions

In this work, a technique for obtaining thin-film coatings of vanadium oxide on various substrates, including quartz glass, sapphire, silicon and optical fibers, has been successfully developed and described. The main practical result is a significant reduction in the time required for studying the deposition and annealing processes of multiphase materials. A detailed comparative analysis of the reduction of multiphase coatings (containing vanadium mainly in the valence state of +5) to single-phase VO_2_(M) by annealing in an atmosphere of hydrogen and argon was carried out. The new result is that conversion to monophase VO_2_(M) in an inert atmosphere occurs due to the formation of elemental carbon as a result of thermal decomposition of organometallic precursors. This finding adds to our understanding of the chemical processes involved in obtaining vanadium oxide from organic precursors by demonstrating that during high-temperature treatments in inert media where both structural reorganization and stoichiometric changes take place, it is important to take into account reductions in carbon content.

VO_2_ films annealed with hydrogen exhibit a narrower phase-transition hysteresis loop (~5 °C) compared to samples annealed with argon (~9 °C), although with a noticeable presence of OH groups. It is important to note that the coatings obtained by both methods demonstrate an increase in transmission in the frequency range of 1 THz and the infrared range when heated by ~50–60%, which correlates with an increase in electrical resistance of ~10^3^–10^4^ times. Argon-annealed coatings are particularly well suited for creating laser modulators, as they avoid significant increases in back reflection when transmission changes due to phase transitions.

## Figures and Tables

**Table 1:** The results of comparing the electrical characteristics of the phase transition of vanadium oxide coatings during annealing in hydrogen and argon. The time of each annealing is 10 min. The thickness of the deposited sample is 250 nm.

Annealing Temperature	Hydrogen Flow	Argon Flow
	R_(40)_/R_(90)_	R_(40)_/R_(90)_
250	1.3	1.8
290	5.4	1.2
320	183	2
350	3150	2.7
380	270	167
410	2.7	3100

**Table 2:** The results of comparing the optical characteristics of the phase transition of vanadium oxide coatings during annealing in hydrogen and argon. The time of each annealing is 10 min. The thickness of the deposited sample is 190 nm.

Annealing Temperature	Hydrogen Flow	Argon Flow
	T_(40)_ − T_(90)_	T_(40)_ − T_(90)_
250	1	1
290	59	8
320	47	27
350	36	49
380	12	39
410	2	30

## Data Availability

The original contributions presented in this study are included in the article. Further inquiries can be directed to the corresponding author.
